# Mapping Lysosomal Storage Disorders with Neurological Features by Cellular Pathways: Towards Precision Medicine

**DOI:** 10.3390/cimb47121009

**Published:** 2025-12-01

**Authors:** Anna Makridou, Evangelie Sintou, Sofia Chatzianagnosti, Sofia Gargani, Maria Eleni Manthou, Iasonas Dermitzakis, Paschalis Theotokis

**Affiliations:** 1 Department of Histology-Embryology, School of Medicine, Aristotle University of Thessaloniki, 54124 Thessaloniki, Greece; annmak4@gmail.com (A.M.); evangelisintou@gmail.com (E.S.); sofiachatzianagnosti@gmail.com (S.C.); sgargani@bio.auth.gr (S.G.); mmanthou@auth.gr (M.E.M.); ptheotokis@auth.gr (P.T.); 2 2nd Department of Neurology University General Hospital AHEPA, Medical School, Aristotle University of Thessaloniki, 54124 Athens, Greece

**Keywords:** lysosomal storage disorders, neurological involvement, enzymatic hydrolytic defects, autophagy, progressive neurological symptoms, precision medicine, cross-organelle interactions, transporter-related defects, biogenesis and signaling

## Abstract

Lysosomal storage disorders (LSDs) represent a diverse group of inherited metabolic diseases in which impaired lysosomal function leads to progressive accumulation of undegraded substrates and widespread cellular dysfunction. Although traditionally classified according to the type of stored macromolecule, this substrate-based approach often fails to reflect the underlying molecular mechanisms. Recent advances in genetics and cell biology have prompted a shift toward functional classifications that group disorders by the lysosomal pathway disrupted—namely, enzymatic hydrolytic defects, transporter-related defects, biogenesis and signaling defects, and cross-organelle interaction abnormalities. This framework better captures disease complexity and provides a translational roadmap for precision medicine. The neurological system, with its high metabolic demands and vulnerability to impaired clearance mechanisms, is particularly affected, leading to clinical phenotypes ranging from developmental delay to severe neurodegeneration. Genomic technologies and multi-omics platforms have facilitated earlier diagnoses, revealed atypical variants, and informed the development of tailored therapies such as enzyme replacement, substrate reduction, chaperone-based approaches, and gene therapy. The current review proposes a cellular-pathway-oriented framework for classifying LSDs with neurological features and underscores how such an approach can assist in the development of personalized therapeutic strategies.

## 1. Introduction

The CNS, though constituting merely 2% of the body mass, is exceptionally vulnerable to disruptions in cellular homeostasis due to its high metabolic demands and limited regenerative capacity. Among the critical organelles ensuring neuronal integrity, lysosomes play a pivotal role in degrading and recycling macromolecules [1]. The lysosomal system comprises membrane proteins, autophagy-related factors, and a wide range of hydrolases that mediate macromolecule degradation. Its activity is tightly regulated to adapt to environmental cues and cellular demands. Deficiencies in lysosomal enzymes or specific membrane proteins result in the accumulation of substrates such as sphingolipids, oligosaccharides, and mucopolysaccharides, ultimately driving cellular dysfunction and death. Neurological involvement is a nearly universal feature of such defects. Beyond these well-established genetic deficiencies, growing evidence indicates that lysosomal dysfunction also contributes to a broad spectrum of common diseases, including neurodegenerative, neoplastic, and metabolic disorders, particularly as age-associated decline in lysosomal function contributes to cellular stress and neurodegeneration [1]. Yet, despite this expanding landscape, lysosomal storage disorders (LSDs) remain paradigmatic models for understanding the molecular and cellular consequences of impaired lysosomal function.

In LSDs, a group of inherited metabolic diseases, the failure of specific lysosomal enzymes or associated trafficking proteins disrupts the major pathways through which lysosomes normally eliminate cellular debris, leading to the accumulation of undegraded substrates within lysosomes and subsequent cellular dysfunction. Endocytosis and phagocytosis continuously deliver extracellular material, such as lipids, glycoproteins, or apoptotic cell remnants, into endosomes and phagosomes that require fusion with functional lysosomes for degradation; in LSDs, incomplete catabolism at this stage results in progressive intralysosomal accumulation (that is, ‘storage’) [2]. Similarly, autophagy—the sequestration of damaged organelles and macromolecules into autophagosomes—depends on efficient lysosomal fusion and enzymatic digestion and is frequently impaired in LSDs, leading to secondary mitochondrial dysfunction and cellular stress. Even macropinocytosis, which imports extracellular fluid and soluble substrates into vesicles that converge on lysosomes, contributes to the pathological buildup when degradative steps are blocked. Collectively, these impaired clearance routes explain the widespread substrate storage that defines LSDs, linking diverse endocytic and autophagic inputs to a common point of failure within the lysosome [3].

Over 70 distinct LSDs have been identified, many of which prominently affect the CNS, manifesting as progressive neurodegeneration, cognitive decline, motor deficits, and seizures [1]. Traditionally, LSDs have been classified according to the nature of the accumulated substrate; however, recent advances in molecular biology and genetics have highlighted the importance of understanding these conditions through the lens of pathogenetic function. This approach organizes disorders based on the specific aspect of lysosomal biology that is disrupted, thereby providing a mapping that more closely reflects disease pathogenesis and therapeutic opportunities. Within this functional classification, four major categories can be distinguished. The first includes enzymatic hydrolytic defects, in which deficiencies of specific lysosomal hydrolases—or their essential cofactors—lead to the accumulation of undegraded macromolecules. The second category encompasses transporter-related defects, caused by impaired lysosomal membrane proteins that regulate the import or export of small molecules such as amino acids, sugars, lipids, or cholesterol. The third group comprises biogenesis and signaling defects, in which lysosomal assembly, enzyme trafficking, or regulatory pathways are disrupted, often resulting in multisystemic manifestations. Finally, cross-organelle interaction defects describe disorders where lysosomal dysfunction perturbs communication with other organelles, such as mitochondria and autophagosomes, leading to broader disturbances in cellular homeostasis [4].

The pathophysiology of LSDs extends beyond substrate accumulation. Secondary mechanisms, such as impaired autophagy, mitochondrial dysfunction, oxidative stress, and inflammation, contribute to neuronal death and disease progression. These insights have propelled research into targeted therapies. Gene silencing therapy (GST) is emerging as a potential alternative for treating LSDs, though current studies remain at the preclinical stage, mainly in sphingolipidoses, glycogen storage disorders, and mucopolysaccharidoses. GST employs diverse approaches, including siRNA, shRNA, antisense oligonucleotides (ASOs), and CRISPR/Cas9, with siRNA being the most widely used. The rapid progress of genome-editing tools and the recent approval of the first ex vivo CRISPR/Cas9 therapy (Casgevy^®^), alongside expanding markets for siRNA and ASO drugs, suggest strong future potential. Nanomedicine is central to advancing GST, offering non-viral nucleic acid delivery systems that can overcome limitations of viral vectors. Lipid nanoparticles (LNPs), already validated in siRNA therapeutics (ONPATTRO™) and COVID-19 vaccines, provide a promising platform, although organ-specific targeting, especially across the blood–brain barrier (BBB), remains a challenge. Strategies such as decorated nanosystems, endogenous lipid modifications, and intranasal administration for CNS delivery are under investigation. Overall, GST addresses unmet clinical needs in LSDs, but further research, improved models, and coordinated scientific efforts are essential to translate these advances into effective, personalized therapies [5]. Furthermore, the heterogeneity of clinical presentations and the rarity of these disorders complicate their diagnosis and management [6].

This review proposes a cellular-pathway-oriented framework for classifying LSDs with neurological involvement, highlighting how such an organizational approach can refine our understanding of LSDs and accelerate the development of personalized therapeutic strategies. Building on this perspective, the review aims to consolidate contemporary knowledge on the molecular, cellular, and therapeutic landscape of LSDs through a functional lens. Achieving this goal will assist in facilitating the translation of emerging technologies into clinically meaningful advances and further promote precision medicine in the field of lysosomal biology.

## 2. Classification of Lysosomal Storage Disorders by Cellular Pathway Dysfunction

**Figure 1:**
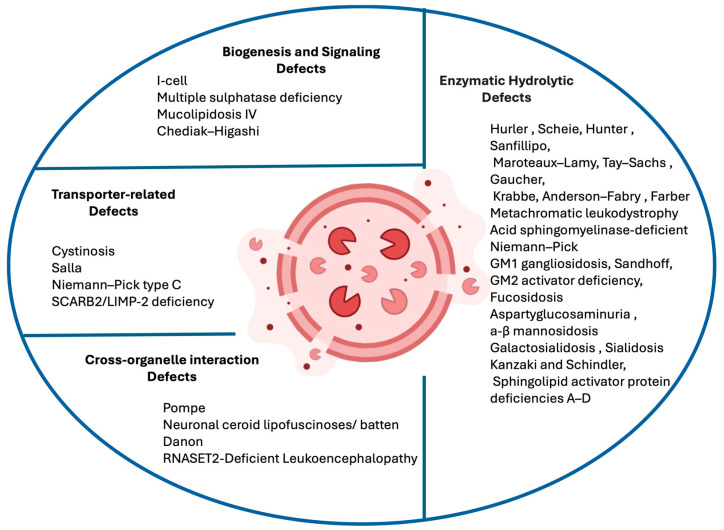
LSDs are grouped according to the disrupted pathway: enzymatic hydrolytic defects (deficient lysosomal hydrolases or cofactors), transporter-related defects (abnormal lysosomal membrane transporters), biogenesis and signaling defects (faulty lysosomal assembly or enzyme trafficking), and cross-organelle interaction defects (impaired lysosome–organelle communication). This framework highlights the diversity of LSDs and supports precision therapeutic approaches. Created with BioRender.com (https://www.biorender.com, accessed on 10 November 2025).

An increasingly influential approach to classification focuses on the lysosomal function that is impaired. This functional perspective aligns with current therapeutic strategies targeting specific cellular pathways. Within this model, disorders can be grouped into four categories as shown in Figure 1: (1) enzymatic hydrolytic defects, where specific lysosomal hydrolases are deficient, preventing the degradation of macromolecules (e.g., mucopolysaccharidoses, sphingolipidoses, glycoproteinoses); (2) transporter-related defects, arising from mutations in lysosomal transport proteins that regulate the import or export of metabolites (e.g., cystinosis, Salla disease, Niemann–Pick type C); (3) biogenesis and signaling defects, involving impaired assembly, maturation, or regulation of lysosomes (e.g., I-cell disease, Hermansky–Pudlak syndrome, Chediak–Higashi syndrome); and (4) cross-organelle interaction defects, where lysosomal dysfunction interferes with other cellular systems such as autophagy, mitochondrial dynamics, or endosomal trafficking. This functional classification strengthens the connection between genotype and phenotype while supporting the identification of therapeutic targets. In the era of precision medicine, such a system reflects the biological underpinnings of disease and facilitates the design of targeted interventions, from enzyme replacement therapy (ERT) and pharmacological chaperones to gene-based therapies.

### 2.1. Enzymatic Hydrolytic Defects

Enzymatic hydrolytic defects represent the largest group of LSDs, as presented in the following Table 1. In these conditions, the primary pathogenic mechanism involves deficiencies of specific lysosomal hydrolases responsible for degrading complex macromolecules such as glycosaminoglycans (GAGs), sphingolipids, and glycoproteins. Without proper enzymatic activity, partially degraded substrates accumulate within lysosomes, leading to cellular dysfunction and progressive tissue damage. Classic examples include the mucopolysaccharidoses, sphingolipidoses, and glycoproteinoses, each of which is defined by the type of substrate that fails to be degraded. In some cases, as with sphingolipid activator protein deficiencies (SAPs), the enzyme itself is intact but lacks an essential cofactor required for activity. This group illustrates the fundamental principle of lysosomal pathology: that a single defective enzyme can disrupt cellular metabolism and cause widespread, multisystemic disease [7].

Hurler syndrome is a genetic LSD caused by mutations in the IDUA gene, leading to a deficiency of the enzyme α-L-iduronidase. This enzyme is essential for breaking down GAGs like heparan sulfate and dermatan sulfate in lysosomes. Without it, GAGs accumulate in cells, causing progressive tissue damage and multisystem dysfunction. Hurler syndrome is the severe phenotype of Mucopolysaccharidosis type I (MPS I), presenting in infancy or early childhood, with progressive skeletal, cardiac, respiratory, ocular, and neurological involvement, leading to premature death without intervention. The mildest form of MPS I is referred to as Scheie syndrome, in which residual enzyme activity slows GAG accumulation, leading to later onset and milder symptoms [8]. Current treatments include ERT and hematopoietic stem cell transplantation (HSCT), though ERT struggles to cross the BBB, limiting its effectiveness in treating neurological symptoms. Gene therapy and improved ERT delivery are being explored to enhance outcomes [9,10].

Similarly, Hunter syndrome, or Mucopolysaccharidosis type II (MPS II), is an X-linked LSD caused by mutations in the IDS gene, which encodes the enzyme iduronate-2-sulfatase (I2S), essential also for breaking down GAGs [11]. A broad spectrum of variants of the IDS has been documented across different countries and ethnic groups, and although disease expression spans a continuum of severity, it is generally categorized into attenuated and severe forms, with the latter accounting for approximately two-thirds of cases. Clinically, deficiency of IDS results in multisystem involvement, leading to a markedly severe phenotype. The most frequent clinical features include organomegaly, cardiorespiratory dysfunction, coarse facial features, skeletal abnormalities with joint stiffness, and growth retardation with short stature. In addition, the severe phenotype is characterized by profound neurological involvement, manifesting as progressive cognitive decline and behavioral disturbances [12].

A comparable mechanism underlies in Sanfilippo syndrome (Mucopolysaccharidosis type III, MPS III) and Maroteaux–Lamy syndrome (Mucopolysaccharidosis type VI, MPS VI). More specifically, Sanfilippo syndrome is an autosomal recessive LSD caused by deficiencies in one of four enzymes involved in heparan sulfate degradation—heparan N-sulfatase (SGSH, type A), α-N-acetylglucosaminidase (NAGLU, type B), acetyl-CoA:α-glucosaminide N-acetyltransferase (HGSNAT, type C), or N-acetylglucosamine-6-sulfatase (GNS, type D) [13]. The overall incidence is estimated at ~1 in 70,000 live births, though rates vary by subtype and geography, with type A being most frequent in Northern Europe, type B in Southern Europe, type C rare, and type D extremely rare. Clinically, all subtypes share a nearly identical phenotype dominated by early-onset, severe, and progressive neurodegeneration with cortical atrophy, progressive dementia, hyperactivity, behavioral disturbances, sleep disruption, motor decline, and profound intellectual disability, while somatic involvement is relatively mild. Pathophysiologically, undegraded heparan sulfate accumulates in lysosomes and other compartments, disturbing lysosomal hydrolase activity, impairing autophagy, disrupting calcium homeostasis, and triggering secondary ganglioside storage, neuronal apoptosis, astrocyte dysfunction, and chronic neuroinflammation, processes compounded by the essential role of heparan sulfate proteoglycans in neurodevelopment and synaptic function. Currently, no curative therapy exists and management is palliative; however, several strategies are under investigation, including ERT (limited by BBB impermeability), substrate reduction (SRT) (e.g., genistein and RNAi targeting HS synthesis), pharmacological chaperones (e.g., glucosamine for HGSNAT), stem-cell-based approaches, and gene therapy using AAV vectors (with promising preclinical results and ongoing clinical trials for subtypes A and B). Despite progress, successful treatment remains challenging, and early diagnosis, along with deeper insights into CNS pathological mechanisms, are critical for future therapeutic breakthroughs [14,15].

On the other hand, Maroteaux–Lamy syndrome (MPS VI), following the same inheritance pattern, is caused by mutations in the ARSB gene, leading to arylsulfatase B deficiency and dermatan sulfate accumulation. Its prevalence varies markedly across populations, ranging from 0.0132 per 100,000 live births in Poland to 7.85 per 100,000 in Eastern Saudi Arabia. Clinically, it is characterized by severe osteoarticular involvement with dysostosis multiplex, short stature, and motor impairment, along with frequent ocular manifestations, Ear, Nose, and Throat (ENT) involvement, and orodental and systemic features, including organomegaly and cardiorespiratory dysfunction. Neurological involvement is generally unusual but may include white matter changes, hydrocephalus, spinal cord compression, carpal tunnel syndrome, and, in some cases, neurocognitive deficits. The clinical presentation spans a continuum from rapidly to slowly progressive forms, rather than discrete categories. ERT with recombinant arylsulfatase B (galsulfase) remains the only approved treatment, significantly reducing urinary GAGs and improving endurance, pulmonary function, and joint mobility, especially when initiated early, though skeletal and ocular disease remain largely refractory. Additional approaches under investigation include gene therapy, SRT, anti-inflammatory strategies, and pharmacological read-through drugs, while the discovery of reliable biochemical and molecular biomarkers is critical for improving early diagnosis, prognosis, and therapeutic monitoring [16,17,18].

Extending the theme of enzymatic failure within the lysosome, Gaucher Disease (GD) is a disorder caused by mutations in the GBA1 gene, which encodes the enzyme β-glucocerebrosidase (β-GC) [18]. This enzyme plays a crucial role in lysosomal metabolism by breaking down glucosylceramide (GluCer) into glucose and ceramide. Mutations in GBA1 lead to β-GC deficiency, resulting in the accumulation of GluCer and its toxic derivative, glucosylsphingosine (GluSph), within lysosomes. This accumulation disrupts normal cellular processes, particularly in macrophages and neurons, leading to progressive systemic and neurological dysfunction. Over 300 mutations in the GBA1 gene have been identified, leading to variable residual enzyme activity and contributing to the phenotypic spectrum of GD. Severe mutations result in minimal to no β-GC activity, characteristic of GD2 [19], while mutations that allow for some residual enzyme function are associated with GD3. Additionally, altered ceramide/GluSer ratios and secondary lipid accumulations, such as cholesterol, further disrupt lysosomal and neuronal homeostasis, exacerbating disease pathology. Thus, mitigating CNS damage in these patients, ongoing research into SRT, gene therapy, and small-molecule approaches is directed toward restoring cellular homeostasis [20,21].

In a similar vein of lysosomal impairment, SAPs A–D are rare autosomal recessive LSDs caused by mutations in the PSAP gene, which encodes the precursor protein prosaposin. Prosaposin is proteolytically cleaved into four sphingolipid activator proteins—saposins A, B, C, and D—that act as non-enzymatic cofactors facilitating degradation of specific glycosphingolipids (GSLs) by their respective enzymes. Pathophysiologically, deficiency of one or more saposins leads to substrate accumulation in lysosomes: for example, SAP-B deficiency causes a phenotype closely resembling metachromatic leukodystrophy (MLD); SAP-C deficiency produces a variant of GD; complete prosaposin deficiency (combined SAP-A–D) leads to widespread neurovisceral storage, multi-sphingolipid accumulation, and severe neurologic and systemic disease. Only a small number of isolated cases of human patients with SAP-B deficiency and prosaposin deficiency have been reported, and, to date, no confirmed human cases of isolated SAP-D deficiency. Clinically, patients present with progressive neurodegeneration, demyelination, often with motor decline, hypotonia, organomegaly, and other visceral involvement depending on which SAP is deficient. There is no specific therapy beyond supportive management; treatment options are very limited and primarily symptomatic [22,23].

In accordance with this model, MLD is a genetic LSD caused primarily by mutations in the ARSA gene. This gene encodes arylsulfatase A (ARSA), an enzyme that plays a crucial role in the breakdown of sulfatides, a type of lipid found in myelin. In some cases, mutations in the PSAP gene, which encodes sphingolipid activator protein B (SAP-B), can also contribute to the disease. The deficiency of ARSA enzyme activity leads to the accumulation of sulfatides in the lysosomes of various cells, particularly in cells of the nervous system. The primary pathological feature of MLD is demyelination caused by the accumulation of sulfatides [24]. In MLD, when ARSA is deficient, sulfatides accumulate in the lysosomes of myelin-producing cells, such as oligodendrocytes in the CNS and Schwann cells in the peripheral nervous system (PNS) [25]. This leads to a loss of myelin integrity and progressive neurological symptoms. Studies have used gene delivery methods, such as viral vectors, to introduce a functional ARSA gene into affected tissues. Some of these gene therapy techniques, including the use of mesenchymal stem cells and combined gene–cell therapies, have shown positive results in preclinical models and are paving the way for future clinical trials [26,27].

In line with this pathogenic mechanism in myelin distribution, Niemann–Pick disease (NPD) is a group of rare LSDs that are characterized by lipid accumulation in various tissues, leading to organ dysfunction and significant neurological symptoms. The disease is caused by mutations in different genes. The two main forms—acid sphingomyelinase-deficient Niemann–Pick disease (ASM-deficient NPD) types A and B and intermediate forms—are both inherited in an autosomal recessive manner and caused by mutations in the SMPD1 gene [28]. The SMPD1 gene is responsible for encoding the enzyme ASM, which plays a critical role in the breakdown of sphingomyelin, a type of lipid that is a key component of cell membranes and myelin sheaths. The enzyme’s function is essential for maintaining cellular lipid balance. Mutations in SMPD1 result in acid sphingomyelinase deficiency (ASMD), causing the accumulation of sphingomyelin in the lysosomes of various tissues, particularly in the liver, spleen, and brain. Type A of ASM-deficient NPD is more severe and manifests with early-onset neurological symptoms, such as developmental delays, motor dysfunction, and neurodegeneration. The disease progresses rapidly, often leading to early childhood death. Type B, in contrast, tends to present with milder or no neurological involvement but significant systemic effects, including organomegaly and pulmonary issues. Some individuals with type B can also experience peripheral neuropathy.

A comparable pattern is seen with Krabbe disease, or globoid cell leukodystrophy. It is a rare autosomal recessive LSD caused by mutations in the GALC gene, which encodes the enzyme galactocerebrosidase. This enzyme plays a crucial role in the breakdown of galactocerebroside, a lipid that is a vital component of the myelin sheath surrounding nerve cells [29]. When the GALC gene is mutated, the galactocerebrosidase enzyme is deficient, leading to the accumulation of psychosine, a toxic lipid that severely disrupts the normal functioning of the nervous system. Mutations in the GALC gene result in a reduced or absent activity of galactocerebrosidase, impairing the breakdown of galactocerebroside and leading to toxic lipid buildup. Interestingly, there is no consistent correlation between the residual GALC enzyme activity and the age of onset of Krabbe disease, meaning that some individuals with slightly higher GALC activity may still develop the disease. This variability in clinical presentation, even within the same family, is likely due to the complex interactions between the mutations present, enzyme activity levels, and other genetic or environmental factors [30].

Adding to this spectrum of defects, Anderson–Fabry disease (AFD), or Fabry disease, is an LSD caused by mutations in the GLA gene. The GLA gene, located on the X chromosome, encodes the enzyme α-galactosidase A (α-GalA), which hydrolyzes the terminal α-galactosyl residues from GSLs, such as ceramide trihexoside or globotriaosylceramide (Gb3). In individuals with AFD, mutations in the GLA gene result in the deficiency or absence of α-GalA enzyme activity, causing a progressive accumulation of Gb3 and its metabolite, globotriaosylsphingosine (Lyso-Gb3), in lysosomes in multiple cell types, including vascular endothelial cells, smooth muscle cells, and neurons [31]. Moreover, Lyso-Gb3, the deacylated form of Gb3, has been identified as a critical catabolite in AFD. It is important in inflammatory responses and fibrosis, further contributing to organ damage. Additionally, Lyso-Gb3 has been linked to mitochondrial dysfunction, autophagy abnormalities, and disruption of the mTOR pathway, which impacts energy metabolism and organ function in AFD patients. Lastly, patients with this disorder present with a variety of symptoms such as pain, strokes, hearing loss, proteinuria, and Chronic Kidney Disease (CKD), as well as cardiac, dermatological, GI, and ophthalmological impairments [32].

**Figure 2:**
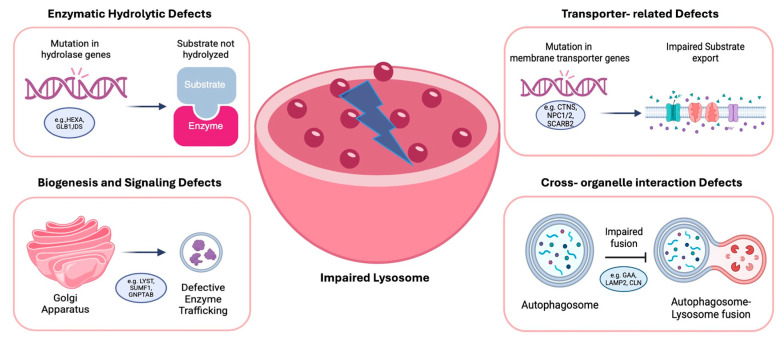
Main cellular pathways in LSDs. Genetic mutations in distinct molecular components of the lysosomal system lead to four mechanistic classes of dysfunction: (1) enzymatic hydrolytic defects, where loss of specific hydrolases causes substrate accumulation; (2) transporter-related defects, impairing metabolite exchange and leading to osmotic and lipid imbalances; (3) niogenesis and signaling defects, involving mis-sorting or defective maturation of lysosomes; and (4) cross-organelle interaction defects, in which lysosomal stress disrupts communication with mitochondria, ER and the autophagy machinery. Created with BioRender.com (https://www.biorender.com, accessed on 10 November 2025).

In line with this model, GM1 gangliosidosis is a disorder primarily caused by mutations in the GLB1 gene, which encodes the enzyme beta-galactosidase. This enzyme plays a crucial role in the breakdown of GM1 gangliosides, a type of GSL that is vital for normal neuronal function. In GM1 gangliosidosis, a deficiency or absence of beta-galactosidase leads to the toxic accumulation of GM1 gangliosides in lysosomes of neurons and other cells [33], thus disrupting normal cellular processes, particularly in the nervous system, and leading to neurodegeneration and organ dysfunction [34,35]. Over 100 distinct mutations have been identified in the GLB1 gene, and these mutations can result in varying levels of enzyme activity, which in turn affects the severity of the disease. Missense and nonsense mutations are the most prevalent types of mutations found in the GLB1 gene, with exons 2, 6, 15, and 16 being common regions where pathogenic mutations are clustered [36]. Lastly, severe mutations that lead to the complete loss of enzyme activity typically result in more severe forms of the disease, such as Type I (infantile) GM1 gangliosidosis, while milder mutations may lead to later-onset forms, such as Type II (late infantile and juvenile) or Type III (adult) GM1 gangliosidosis [37,38]. Each category’s pathogenesis is summarized in Figure 2.

In line with this shared pathogenic mechanism, in Galactosialidosis, a rare autosomal recessive LSD, cathepsin A regulates and stabilizes β-galactosidase, encoded by the GLB1 gene, and neuraminidase, encoded by the NEU1 gene, in the lysosomal multienzyme complex. This disorder is caused by mutations in the CTSA gene encoding cathepsin A, also known as protective protein/cathepsin A (PPCA). Fewer than 120 cases have been reported worldwide, with only 23 different mutations identified to date, including missense, frameshift, and rearrangements, but until recently, almost no nonsense mutations. Clinical presentation is heterogeneous and broadly classified into early infantile, late infantile, and juvenile/adult forms, with severity and onset correlating to residual CTSA activity. The early infantile phenotype is the most severe, often presenting with fetal hydrops, visceromegaly, cardiac involvement, coarse facial features, skeletal abnormalities, and early death; the late infantile form features growth retardation, dysostosis multiplex, organomegaly, angiokeratoma, hearing loss, and cognitive decline; while the juvenile/adult form, the most common, typically manifests with progressive neurologic impairment, myoclonus, seizures, ataxia, vision loss, angiokeratoma, and cardiomyopathy. MRI findings may show cerebral and cerebellar atrophy. Mutational studies reveal frequent clustering at conserved residues, with genotype–phenotype correlation supported by in silico structural modeling. PPCA’s dual catalytic and protective roles likely explain the multisystemic manifestations, as defects not only disrupt GLB1 and NEU1 activity but may also impair esterase/deaminase functions in platelets, endothelial cells, and the cardiovascular system, broadening the phenotypic spectrum. Currently, no curative therapy exists, though HSCT and ERT remain theoretical possibilities, and detailed structural studies of CTSA and its protein–protein interactions may open future therapeutic avenues [39].

A parallel pathway to lysosomal dysfunction can be found in Sialidosis, a rare autosomal recessive LSD caused by mutations in the NEU1 gene, leading to deficiency of lysosomal neuraminidase. The disease is classified into two main types. Type I, the milder form, typically presents in the second or third decade of life with myoclonus, gait abnormalities, visual impairment, and sometimes slight cognitive deficits, while physical development is usually normal. Type II is more severe and subdivided into congenital, infantile, and juvenile forms, featuring hepatosplenomegaly, dysostosis multiplex, coarse facial features, cherry-red spots, and progressive neurological impairment. Pathologically, accumulation of sialyloligosaccharides occurs in neurons and other tissues, and neuroimaging may reveal cerebellar and cortical atrophy. Clinical severity generally correlates with residual NEU1 enzyme activity. Therapeutic approaches are limited, with research exploring ERT, chaperone therapy, and gene therapy in animal models. Due to the rarity of sialidosis, studies are relatively scarce, and most knowledge derives from case reports and experimental models [40,41].

Venturing into another distinct category of LSDs, Farber syndrome is a rare autosomal recessive disorder caused by mutations in the ASAH1 gene, which encodes the enzyme acid ceramidase. This enzyme plays a crucial role in the breakdown of ceramide, a type of sphingolipid, into sphingosine and free fatty acids. When mutations occur in the ASAH1 gene, the enzyme activity is deficient or absent, leading to the accumulation of ceramide in various tissues, including the nervous system. In normal cells, acid ceramidase helps to break down ceramide into its metabolites, sphingosine and fatty acids, which are essential for normal cellular functions, including cell membrane formation and signaling [42]. However, in individuals with Farber syndrome, due to the ASAH1 gene mutation, this breakdown is impaired, and ceramide accumulates in the lysosomes of cells throughout the body, resulting in progressive tissue damage, particularly in the nervous system, musculoskeletal system, and respiratory tract [43,44,45].

Another illustrative example of the LSD spectrum is Fucosidosis, a rare autosomal recessive disorder caused by mutations in the FUCA1 gene, leading to a deficiency of the enzyme α-L-fucosidase. This deficiency prevents the proper degradation of fucosylated glycoproteins and glycolipids, resulting in their accumulation in multiple tissues, including the brain, liver, bone, and skin. The disease manifests with progressive neurological deterioration, growth retardation, coarse facial features, hepatosplenomegaly, skeletal abnormalities, angiokeratomas, epilepsy, and recurrent respiratory infections. Onset and severity vary, with early-onset (type I) cases progressing rapidly and often leading to death in childhood, whereas later-onset (type II) cases progress more slowly, sometimes allowing survival into early adulthood. Diagnosis is confirmed by identifying FUCA1 mutations and measuring α-L-fucosidase activity, often supported by characteristic neuroimaging findings. Treatment is primarily supportive, but HSCT, gene therapy, and ERT show promise if applied early, although access and feasibility are limited by the rarity of the disease and socioeconomic factors [46].

Similarly, classified among LSDs is Aspartylglucosaminuria (AGU). It is inherited in an autosomal recessive pattern and is caused by mutations in the AGA gene, leading to deficient aspartylglucosaminidase activity and impaired degradation of glycoproteins. Worldwide, over 30 AGA variants have been identified, with the disease most frequently reported in the Finnish population due to a founder effect. Pathogenic variants result in the accumulation of glycosaminoglycans, which are toxic to cells over time. Clinically, AGU presents with early developmental delays, progressive intellectual disability, autistic features, skeletal and connective tissue abnormalities, macrocephaly, hernias, recurrent respiratory and ear infections, gait disturbances and seizures. Neuroimaging often reveals cerebral and cerebellar atrophy, white matter abnormalities, and thalamic hypointensities, reflecting progressive neurological disease. There is currently no curative treatment; management is supportive and focuses on symptomatic care. Preclinical studies of ERT in AGA-null mice show promise, but human trials have not yet been initiated. Early genetic diagnosis will be crucial for the future success of potential disease-modifying therapies [47,48].

Another representative lysosomal disorder is α-Mannosidosis, a rare autosomal recessive disease caused by pathogenic variants in the MAN2B1 gene, encoding lysosomal α-mannosidase. Affected children are typically born without obvious signs, with symptoms emerging progressively and spanning a clinical spectrum now categorized into three types: type 1 (mild, onset after 10 years, slow progression, no skeletal involvement), type 2 (moderate, onset before 10 years, skeletal abnormalities, progressive ataxia by the second to third decade; the most common form), and type 3 (severe, neonatal/infantile onset, rapid progression, skeletal disease, CNS involvement, and early death). Diagnosis is based on deficient lysosomal α-mannosidase activity combined with elevated urinary mannose-rich oligosaccharides, confirmed by MAN2B1 sequencing. To date, genotype–phenotype correlation remains poor, consistent with other lysosomal disorders. Natural history studies show progressive motor and neurological decline, reduced lung function, and impaired endurance correlating with urinary oligosaccharide excretion, which serves as a potential biomarker. Therapeutic strategies include HSCT, which can normalize enzyme activity, stabilize cognition, improve hearing, reduce infections, and ameliorate skeletal and somatic features, with the best outcomes when performed early. ERT with recombinant human α-mannosidase (velmanase alfa, lamzede) has been approved in Europe for mild–moderate disease, demonstrating reductions in serum oligosaccharides and improvements in motor function and quality of life in phase I–III trials, though efficacy in CNS disease is limited as the recombinant enzyme does not cross the BBB. Future directions include pharmacological chaperone therapy, which could address neurological manifestations by stabilizing misfolded enzymes and potentially reaching the CNS [49].

Consistent with this pathogenic framework, β-Mannosidosis is a rare autosomal recessive LSD caused by deficiency of the lysosomal enzyme β-mannosidase (EC 3.2.1.25), which cleaves β-linked mannose residues in N-linked glycoproteins. Clinical manifestations vary widely in severity and age of onset, with common features including intellectual disability, speech impairment, hearing loss, hypotonia, epilepsy, and peripheral neuropathy. The disorder results in accumulation of specific oligosaccharides, primarily the disaccharide Man(β1–4)GlcNAc and its derivatives, in tissues and urine, sometimes including sialylated trisaccharides due to abnormal sialylation. The human β-mannosidase gene (MANBA) is located on chromosome 4q22–25, and pathogenic variants identified include homozygous missense and splice-site mutations leading to abnormal mRNA and truncated proteins. β-Mannosidosis is biochemically diagnosed by markedly reduced lysosomal β-mannosidase activity, with residual activity possibly contributed by other mannosidases. Because of the rarity and phenotypic variability, few patients have been studied; thus, diagnosis can be challenging, with no curative therapy established [50].

Extending the spectrum, Kanzaki disease is a disorder caused by mutations in the NAGA gene, which encodes the lysosomal enzyme α-N-acetylgalactosaminidase (α-NAGA). This enzyme is responsible for the degradation of specific glycopeptides, particularly the Tn-antigen (GalNAcα1-O-Ser/Thr)**,** a core structure in O-linked glycoconjugates. Loss of α-NAGA function leads to intracellular accumulation of undegraded substrates, particularly within lysosomes, thus defining it as a lysosomal impairment disorder. Structural modeling of mutant α-NAGA proteins, particularly the R329W and R329Q variants found in Kanzaki disease, reveals conformational changes that compromise enzyme stability and function. Immunocytochemical analysis confirms lysosomal accumulation of Tn-antigen in patient fibroblasts. Clinically, Kanzaki disease presents in adulthood with milder symptoms than Schindler disease (its severe infantile counterpart) and may include angiokeratomas, mild cognitive decline, hearing loss, and lymphadenopathy. The disease exhibits a unique genotype–phenotype paradox, with certain mutations causing unexpectedly mild or severe phenotypes independent of predicted enzymatic activity levels [51].

A further lysosomal defect is seen in Tay–Sachs disease, an autosomal recessive disorder caused by mutations in the HEXA gene, which encodes the α-subunit of β-hexosaminidase A (HexA) [52]. This enzyme is crucial for the degradation of GM2 gangliosides, a type of lipid found in neuronal cell membranes. Mutations in HEXA lead to HexA deficiency, resulting in the accumulation of GM2 gangliosides within the lysosomes of nerve cells, ultimately causing progressive neurodegeneration [53]. The severity of Tay–Sachs disease correlates with the residual HexA enzymatic activity, which varies depending on the specific HEXA mutation. The most severe form, the infantile type, manifests in early infancy and leads to rapid neurological decline [54]. Juvenile and late-onset forms present with milder symptoms, often appearing in adolescence or adulthood. Gene therapy strategies involve using adeno- or adeno-associated viral vectors to deliver functional HEXA cDNA, aiming to restore HexA activity. These approaches are being evaluated in animal models, including HEXA-deficient mice and Jacob sheep, which naturally develop Tay–Sachs disease with similar pathological features to humans. Ongoing research focuses on developing targeted therapies to prevent GM2 ganglioside accumulation and mitigate neuroinflammation, offering potential future treatments for this devastating genetic disorder [55,56].

In accordance with this model, GM2 activator deficiency syndrome, also known as the AB variant of GM2 gangliosidosis, is a rare and severe LSD caused by mutations in the GM2A gene [56] and is inherited in the same pattern [57,58]. This gene encodes the GM2 activator protein, which plays a critical role in the breakdown of GM2 gangliosides within lysosomes. More specifically, this protein is essential for the hydrolysis of GM2 gangliosides by the enzyme complex Hex A. For effective breakdown, GM2 activator protein acts as a cofactor for Hex A. Mutations in the GM2A gene impair the function of the activator protein, preventing it from assisting in the proper degradation of GM2 gangliosides. As a result, a deficiency of this protein leads to the accumulation of GM2 gangliosides, particularly in the CNS, causing toxic effects in neurons and leading to severe neurological impairments.

A similar convergence occurs in Sandhoff syndrome, which is a rare LSD inherited in the same manner [59], primarily affecting the nervous system and caused by mutations in the HEXB gene. This gene encodes the enzymes beta-hexosaminidase A and B, which are important in the lysosomal degradation pathway that helps maintain cellular function by processing and recycling lipids such as GM2 gangliosides and globosides. When mutations occur in the HEXB gene, these enzymes become deficient or absent, leading to an impairment in the degradation process and thus, a buildup of these lipids in the lysosomes, particularly in nerve cells. This accumulation causes neurodegeneration, resulting in severe neurological impairment in patients with this syndrome.

**Table 1:** Overview of lysosomal disorders with neurological impairment caused by enzyme hydrolytic defects.

Enzymatic Hydrolytic Defects	Gene(s)	Protein(s)	Clinical Features	Inheritance	Prevalence	References
Hurler Syndrome	IDUA	Alpha-L-iduronidase	Skeletal deformities, cardiopathy, hepatosplenomegaly, corneal clouding, CNS involvement, hearing loss, short stature	Autosomal recessive	1:100,000	[10]
Scheie Syndrome	IDUA	Alpha-L-iduronidase	Skeletal anomalies, cardiopathy, hepatosplenomegaly, corneal clouding, hearing loss, stable condition	Autosomal recessive	0.07:100,000	[60]
Hunter Syndrome	IDS	Iduronate 2-sulfatase	Skeletal deformities, hernias, hepatosplenomegaly, cardiopathy, corneal clouding, hearing loss, joint stiffness, growth delay, sleep apnea, hyperactivity	X-linked recessive	0.38–1.09 :100,000	[10,11,61]
Sanfillipo Syndrome	SGSH, NAGLU, HGSNAT, GNS	N-sulfoglucosamine sulfohydrolase, N-acetyl-alpha-glucosaminidase, Heparan-alpha-glucosaminide N-acetyltransferase, Glucosamine (N-acetyl)-6-sulfatase	Neurocognitive decline, behavioral dysregulation, speech regression, coarse facies, hernias, skeletal and visceral involvement, hearing loss, growth delay	Autosomal recessive	0.76:100,000	[62]
Maroteaux– Lamy Syndrome	ARSB	Arylsulfatase B	Coarse facies, skeletal and joint anomalies, corneal clouding, hearing loss, dental dysplasia, organomegaly, growth delay, GI issues, skin thickening, connective tissue defects	Autosomal recessive	0.12:100,000	[17]
Gaucher Disease	GBA1	Glucosylceramidase beta 1	Hepatosplenomegaly, cytopenias, skeletal anomalies, fatigue, coagulopathy, anemia, thrombocytopenia, seizures, movement disorders, growth delay	Autosomal recessive	0.45– 25.0:100,000	[18]
Sphingolipid activator protein deficiencies A–D	PSAP	Prosaposin	Lysosomal storage disorder, skeletal anomalies, neurological involvement, motor and cognitive impairment, cherry-red macula	Autosomal recessive	No data available	[22]
Metachromatic leukodystrophy	ARSA	Arylsulfatase A	Motor impairment, stiffness, hypotonia, seizures, regression, optic atrophy, dysphagia, behavioral changes, urinary incontinence, psychiatric features	Autosomal recessive	0.16– 1.85: 100,000	[27]
Acid sphingomyelinase-deficient Niemann–Pick disease	SMPD1	Sphingomyelin phosphodiesterase 1	Hepatosplenomegaly, developmental delay, cherry-red spot, motor decline, spasticity, dysphagia	Autosomal recessive	0.5–1:100,000	[63]
Krabbe Disease	GALC	Galactosylceramidase	Irritability, dysphagia, hypotonia, spasticity, motor regression, optic atrophy, hearing loss, seizures, developmental decline, fever, macrocephaly, peripheral neuropathy	Autosomal recessive	1:310,000	[30]
Anderson–Fabry Disease	GLA	Galactosidase alpha	Acroparesthesias, angiokeratomas, anhidrosis, corneal opacities, GI symptoms, cardiomyopathy, arrhythmias, valvular disease, stroke, hearing loss, CNS involvement, lymph hypertrophy, renal impairment	X-linked Recessive	0.5% 61	[31]
GM1 gangliosidosis	GLB1	Galactosidase beta 1	Growth delay, hypotonia, macrocephaly, hepatosplenomegaly, seizures, spasticity, cherry-red spot, motor impairment, dysphagia, psychiatric symptoms	Autosomal recessive	No data available	[38]
Galactosialidosis	CTSA	Cathepsin A	Hepatosplenomegaly, coarse facies, dysostosis multiplex, joint stiffness, cardiopathy, developmental delay, seizures, visual impairment, ataxia, muscle weakness, cherry-red spot	Autosomal recessive	120 cases reported	[39]
P-Sialidosis	NEU1	Neuraminidase 1	Hepatosplenomegaly, dysostosis multiplex, coarse facies, skeletal anomalies, developmental delay, ophthalmologic issues, recurrent infections, hearing loss, ataxia, psychiatric symptoms, renal dysfunction	Autosomal recessive	1:4,200,000	[41]
Farber Syndrome	ASAH1	N-acylsphingosine amidohydrolase 1	Joint deformities, subcutaneous and nodular skin lesions, hoarseness, lipogranulomas, pain, feeding issues, growth delay, hepatosplenomegaly, neurologic involvement	Autosomal recessive	No data available	[43]
Fucosidosis	FUCA1	Alpha-L-fucosidase 1	Developmental delay, hypotonia, coarse facies, dysostosis multiplex, skeletal anomalies, organomegaly, cardiopathy, recurrent infections, hearing loss, seizures, speech and behavioral abnormalities, neurologic decline	Autosomal recessive	1:200,000	[46]
Aspartylglucosaminuria	AGA	Aspartylglucosaminidase	Language delay, behavioral changes, motor delay, coarse facies, joint stiffness, hepatosplenomegaly, recurrent infections, hypotonia, orthopedic issues, dental anomalies, seizures, neurologic decline	Autosomal recessive	1:18,500	[48]
A-mannosidosis	MAN2B1	Mannosidase alpha class 2B member 1	Developmental delay, hearing loss, coarse facies, skeletal and joint anomalies, hepatosplenomegaly, dental anomalies, GI dysfunction, immunodeficiency, muscle weakness, ataxia, seizures	Autosomal recessive	1:600,000–1:1,000,000	[49]
β-mannosidosis	MANBA	Mannosidase beta	Developmental delay, coarse facies, skeletal anomalies, hepatosplenomegaly, dental anomalies, ataxia, seizures, visual impairment, muscle weakness, immunodeficiency, behavioral changes	Autosomal recessive	No data available	[50]
Kanzaki and Schindler Disease	NAGA	Alpha-N-acetylgalactosaminidase	Severe neurodevelopmental delay, hypotonia, seizures, neuroregression, hearing loss, visual impairment, macrocephaly, coarse facial features	Autosomal recessive	No data available	[51]
Tay–Sachs	HEXA	Hexosaminidase subunit alpha	Growth delay, hypotonia, hyperacusis, seizures, motor dysfunction, dysphagia, cherry-red spot, blindness, spasticity, macrocephaly, respiratory compromise	Autosomal recessive	1:3500	[52]
GM2 activator deficiency	GM2A	Ganglioside GM2 activator	Growth delay, hypotonia, seizures, motor regression, spasticity, optic atrophy, hearing loss, cherry-red spot, hepatosplenomegaly, dysphagia, neurologic decline	Autosomal recessive	No data available	[57]
Sandhoff syndrome	HEXB	Hexosaminidase subunit beta	Growth delay, hypotonia, macrocephaly, seizures, spasticity, muscle weakness, dysphagia, motor dysfunction, cherry-red spot	Autosomal recessive	No data available	[59]

GI, Gastrointestinal; CNS, Central Nervous System. All protein names and gene symbols were curated and verified using the Human Protein Atlas database [64].

### 2.2. Transporter-Related Defects

Transporter-related defects are caused by abnormalities in lysosomal membrane proteins that regulate the import and export of small molecules. Lysosomes rely on specialized transporters to maintain homeostasis by moving metabolites, degradation products, and ions across their limiting membrane. Mutations in these transporters prevent the efficient clearance or distribution of substrates, resulting in their pathological accumulation. Representative disorders, as shown in Table 2, include cystinosis, where cystine crystals accumulate due to a defective cystine transporter; Salla disease, characterized by impaired sialic acid transport; and Niemann–Pick type C, in which defective NPC1 or NPC2 proteins disrupt intracellular cholesterol and lipid trafficking. Unlike enzymatic defects, where the problem lies in substrate breakdown, transporter-related disorders reflect a failure of lysosomal communication with the cytoplasm and other organelles, highlighting the critical role of lysosomal transport in cellular metabolism [61,62].

Cystinosis is a rare autosomal recessive LSD caused by mutations in the CTNS gene (17p13.2), encoding cystinosin, a cystine–proton co-transporter. Defective transport results in cystine accumulation and crystal formation within lysosomes. Severe biallelic truncating variants typically cause the infantile nephropathic form, while at least one milder allele is associated with juvenile or ocular forms [65]. Clinically, cystinosis is systemic and progressive, with renal Fanconi syndrome leading to end-stage renal disease and multi-organ involvement affecting the eyes, endocrine system, muscles, and the nervous system. Neurological features include hypotonia, tremor, and cognitive and visual–spatial impairment, with MRI showing early white matter changes. Cysteamine remains the only disease-specific treatment, while new delayed-release formulations improve compliance, and experimental strategies such as stem cell transplantation and therapies targeting inflammation and autophagy are under investigation [66,67].

Similarly, Salla Disease is a rare autosomal recessive disorder caused by pathogenic variants in the SLC17A5 gene, which encodes sialin, a lysosomal membrane transporter of sialic acid. Defective sialin leads to the accumulation of free sialic acid in lysosomes and results in a clinical spectrum ranging from the severe infantile free sialic acid storage disease with global developmental delay, coarse facial features, hepatosplenomegaly, and early death, to the milder Salla disease characterized by normal appearance, mild cognitive dysfunction, spasticity, and motor disability. An intermediate severe form presents with hypotonia, hypomyelination, and moderate to severe developmental delay. Diagnosis is confirmed by sequencing of SLC17A5, while elevated urinary free sialic acid may provide an initial clue but is not always reliable. Only a few clinical and imaging studies of Salla disease have been conducted, and the rarity of reported cases highlights the limited knowledge about this disorder [68].

Further supporting this concept, unlike ASM-deficient NPD, Niemann–Pick disease type C (NPC) is caused by mutations in either the NPC1 gene or the NPC2 gene, both of which are involved in the transport of cholesterol and other lipids within lysosomes. NPC1 encodes a 1278-amino-acid transmembrane protein with 13 transmembrane and sterol-sensing domains, while NPC2 is a 151-amino-acid soluble lysosomal protein; deficiencies in either of them lead to accumulation of unesterified cholesterol, sphingosine, and sphingolipids in late endosomes/lysosomes, disrupted lysosomal calcium homeostasis, and impaired endocytic trafficking [69]. Over 300 NPC1 mutations have been reported, mostly private, with nonsense and frameshift mutations correlating with severe, early-onset neurological disease, whereas certain missense mutations, such as the common p.I1061T, are associated with later onset, slowly progressing forms; genotype–phenotype correlations in NPC2 are limited due to the small number of reported cases [63]. This leads to progressive neurological degeneration, characterized by ataxia, cognitive decline, and seizures. Both forms of NPDs are associated with significant neurological and visceral damage, underlining the importance of early diagnosis and ongoing research into potential therapies [70].

Lastly, SCARB2/LIMP-2 deficiency is an autosomal recessive disorder caused by mutations in the SCARB2 gene, which encodes lysosomal integral membrane protein-2 (LIMP-2), a receptor essential for trafficking β-GC into lysosomes and maintaining lysosomal membrane integrity. Loss of function results in lysosomal dysfunction with impaired GluCer degradation, leading to neuronal damage, demyelinating neuropathy, and renal tubular dysfunction. The disease is extremely rare, with only a limited number of cases reported worldwide, most often described as action myoclonus–renal failure (AMRF) syndrome. Symptoms typically present in adolescence or early adulthood and include progressive action myoclonus, seizures, ataxia, and often proteinuria progressing to renal failure, while some patients develop epilepsy without renal disease. Currently, therapy is mostly supportive [66,67].

**Table 2:** Lysosomal disorders categorized by transporter-related defects.

Transporter-Related Defects	Gene(s)	Protein(s)	Clinical Features	Inheritance	Prevalence	References
Cystinosis	CTNS	Cystinosin	Renal pathology, polyuria, polydipsia, growth retardation, dehydration, hypophosphatemic rickets, corneal crystalline deposits, myopathy, endocrine dysfunction, CNS involvement, pancreatic insufficiency.	Autosomal recessive	1:115,000–1:260,000	[67]
Salla	SLC17A5	Solute carrier family 17 member 5	Developmental delay, intellectual disability, motor deficits, hypotonia, speech and language delay.	Autosomal recessive	No data available	[68]
Niemann–Pick disease type C	NPC1, NPC2	NPC intracellular cholesterol transporter 1, NPC intracellular cholesterol transporter 2	Progressive neurological degeneration (ataxia, cognitive decline), seizures	Autosomal recessive	0.5–1:100,000	[63]
SCARB2/LIMP-2 deficiency	SCARB2	Scavenger receptor class B member 2	Epilepsy, ataxia, dystonia, tremor, action myoclonus, renal failure, proteinuria, cardiopathy.	Autosomal recessive	No data available	[71]

CNS, Central Nervous System. All protein names and gene symbols were curated and verified using the Human Protein Atlas database [64].

### 2.3. Biogenesis and Signaling Defects

Biogenesis and signaling defects involve errors in the assembly, maturation, or regulation of lysosomes themselves. In these disorders, the pathogenic mechanism is not the lack of a single degradative enzyme but rather a failure in the processes that deliver enzymes, build functional lysosomes, or regulate lysosomal activity. A prototypical example is I-cell disease (mucolipidosis II, ML II), where defective trafficking prevents lysosomal hydrolases from reaching the organelle, resulting in secondary accumulation of multiple substrates. Other examples include, as illustrated in Table 3, Chediak–Higashi syndrome, which is characterized by abnormalities in LROs, and multiple sulfatase deficiency (MSD), in which impaired enzyme activation leads to combined hydrolase deficiencies. These disorders underscore the complexity of lysosomal biology, demonstrating that proper lysosomal function requires not only intact enzymes but also accurate assembly and regulatory signaling [68,72].

One of the most representative examples of this category is ML II. It is a very rare LSD with autosomal recessive inheritance, caused by mutations in the GNPTAB gene (12q23.3), which encodes the enzyme N-acetylglucosamine-1-phosphotransferase. This enzyme is required for the addition of the mannose-6-phosphate recognition marker to lysosomal enzyme precursors, a critical step for their trafficking to lysosomes. Loss of this targeting signal results in misrouting of lysosomal enzymes, impaired degradation of cellular substrates, and widespread tissue pathology. Clinically, ML II manifests from early infancy with low birth weight, severe growth retardation, coarse facial features, gingival hypertrophy, joint contractures, and skeletal deformities. To date, approximately 200 pediatric cases have been reported worldwide. ML II is invariably progressive and associated with severe morbidity and reduced life expectancy. No curative treatment is available, and bone marrow transplantation (BMT) has been attempted in a small number of children, with limited success in slowing cardiopulmonary progression and allowing partial neurodevelopmental improvement, though outcomes remain poor overall [73,74].

Beyond this example, MSD is an autosomal recessive LSD caused by pathogenic variants in the SUMF1 gene, which encodes the formylglycine-generating enzyme (FGE). FGE is responsible for the post-translational activation of all sulfatases, and its deficiency leads to global sulfatase insufficiency with accumulation of various sulfated compounds, including mucopolysaccharides and sphingolipids. MSD presents with a combination of clinical features seen in individual sulfatase deficiencies, such as developmental regression, hypotonia, ichthyosis, and multi-organ involvement. The disease is classified into neonatal, late infantile, and juvenile forms based on age of onset and severity, with neonatal-onset MSD being associated with the most severe course and reduced survival. Although residual sulfatase activity and genotype may partially influence disease severity, clinical outcomes are not consistently predicted by these factors, likely due to the modifying effects of protein stability, FGE interactions, and other yet unknown cellular mechanisms. There are currently no disease-modifying therapies or interventional clinical trials, though gene therapy and other approaches may become available in the future [75,76].

Further illustrating the critical and diverse roles of the lysosome, in Mucolipidosis Type IV (ML IV), an autosomal recessive LSD, mutations are caused in the MCOLN1 gene (chromosome 19p13.2-p13.3), which encodes the lysosomal TRPML1 channel. This mutation leads to a deficiency that impairs lysosomal Ca^2+^ signaling, trafficking, and autophagy, leading to lipid/protein accumulation, neurodevelopmental delay, retinal degeneration, and gastric achlorhydria. Symptoms include early psychomotor delay, hypotonia progressing to spasticity, severe vision loss, feeding issues, and progressive neurodegeneration. No curative therapy exists; management is supportive, though MCOLN1 gene therapy shows promise in preclinical models [77].

Lastly classified among lysosomal diseases is Chediak–Higashi Syndrome (CHS). It is a rare autosomal recessive disorder caused by mutations in the LYST gene, which encodes a large protein essential for vesicular trafficking and lysosome-related organelle (LRO) function. Mutations—most often private nonsense, missense, frameshift, or splice variants distributed throughout the gene—lead to loss of function, resulting in enlarged lysosomes, abnormal melanosomes, platelet dense bodies, and lytic granules. Clinically, CHS is characterized by partial oculocutaneous albinism, bleeding tendency, immune dysfunction with recurrent infections, progressive neurodegeneration, and a high risk of developing hemophagocytic lymphohistiocytosis (HLH), the main cause of mortality. Neurologic features evolve from developmental and behavioral problems in childhood to progressive degeneration in adolescence and adulthood, including peripheral neuropathy, cerebellar dysfunction, spasticity, Parkinsonism, and cerebral/cerebellar atrophy. A genotype–phenotype correlation has been noted, with truncating variants usually causing severe disease and missense variants associated with milder presentations, although exceptions occur. HSCT can correct hematologic and immunologic defects and improve HLH outcomes if performed early, but it does not halt neurodegeneration. Current therapy is otherwise symptomatic, with supportive neurologic and rehabilitative care; experimental approaches such as gene therapy remain limited due to the large size of LYST [78].

**Table 3:** Lysosomal disorders with neurological impairment associated with biogenesis and signaling defects.

Biogenesis and Signaling Defects	Gene(s)	Protein(s)	Clinical Features	Inheritance	Prevalence	References
I-cell disease	GNPTAB	N-acetylglucosamine-1-phosphate transferase subunits alpha and beta	Dysostosis, short stature, coarse facies, hypertelorism, gingival hypertrophy, cardiac disease, organomegaly, developmental delay, dysmorphic features, inguinal hernia, urinary oligosacchariduria.	Autosomal recessive	200 cases reported	[75]
Multiple sulphatase deficiency	SUMF1	Sulfatase-modifying factor 1	Neurodevelopmental regression, hypotonia, spasticity, seizures, dystonia, motor decline, sensory impairment, organomegaly, skeletal anomalies, optic atrophy, cherry-red macula.	Autosomal recessive	1:1,400,000	[79]
Mucolipidosis IV	MCOLN1	Mucolipin TRP cation channel 1	Dysostosis, facial dysmorphism, organomegaly, cardiopathy, corneal clouding, hernias, seizures, hypotonia, growth retardation, hearing loss, urinary oligosacchariduria.	Autosomal recessive	1:40,000	[77]
Chediak–Higashi	LYST	Lysosomal trafficking regulator	Hypopigmentation, silvery hair, light-colored irides, recurrent infections, leukocyte inclusions, peripheral neuropathy, ataxia, pancytopenia, lymphohistiocytic infiltration, photosensitivity.	Autosomal recessive	No data available	[78]

All protein names and gene symbols were curated and verified using the Human Protein Atlas database [64].

### 2.4. Cross-Organelle Interaction Defects

This category encompasses disorders arising from disrupted communication between lysosomes and other cellular organelles, such as mitochondria, the endoplasmic reticulum, and autophagosomes [4]. Lysosomes are now recognized as central hubs of cellular signaling and metabolic regulation, and defects in their crosstalk with partner organelles have profound consequences for cell survival. For example, Pompe disease results from defective glycogen degradation that also impairs autophagic flux in muscle cells; Danon disease reflects abnormalities in the lysosome–autophagy interface due to LAMP2 deficiency; and the neuronal ceroid lipofuscinoses (NCLs) involve defective clearance of lipopigments with secondary mitochondrial dysfunction. Disorders such as RNASET2-related disease further highlight the role of lysosomes in lipid and RNA metabolism, respectively. By framing these conditions as disturbances of inter-organelle communication, as summarized in Table 4, this category emphasizes the lysosome’s role as a dynamic regulator of cellular homeostasis rather than a passive degradative compartment [61,77,79].

In agreement with this pathogenic framework, Pompe disease is a metabolic disorder caused by mutations in the GAA gene on chromosome 17q25.2–q25.3, leading to deficiency of acid α-glucosidase (GAA) and impaired lysosomal glycogen degradation. The wide spectrum of phenotypes arises from the degree of residual enzyme activity, reflecting the mutational heterogeneity of the GAA gene, which includes missense, nonsense, splicing variants, small deletions/insertions, and complex rearrangements. Infantile-onset Pompe disease (IOPD) is most often associated with severe or null mutations that abolish GAA expression, producing little to no residual activity. Clinically, this form manifests within the first year of life with hypertrophic cardiomyopathy, generalized hypotonia, respiratory failure, and, increasingly recognized with prolonged survival, CNS involvement due to glycogen storage in neurons and glial cells. By contrast, late-onset Pompe disease (LOPD) typically results from milder missense or splicing mutations, such as the common c.-32-13T>G variant, which allow partial enzyme activity. LOPD usually emerges in childhood or adulthood, presenting with slowly progressive proximal muscle weakness, paraspinal involvement, and respiratory insufficiency, while cardiac disease and overt CNS impairment are minimal or absent. Disease progression and severity in both forms are further shaped by compound heterozygosity and genetic modifiers such as the c.510C>T variant, accounting for the considerable variability in age at onset, residual enzyme activity, and clinical course [80,81].

Further supporting this concept, NCL refers to a group of LSDs caused by mutations in CLN1–CLN14, leading to the accumulation of lipopigments (ceroid and lipofuscin) in neurons. To date, 13 autosomal recessive and one autosomal dominant gene variant associated with NCL have been identified. These genes encode diverse proteins, many of which are lysosomal enzymes or transmembrane proteins localized to lysosomes or other cellular organelles, although their exact functions are not fully understood. NCLs are typically marked by progressive vision loss, cognitive and motor decline, seizures, and premature death, with rare adult-onset cases presenting primarily with dementia. The severity and age of onset of symptoms can differ depending on the specific mutation. A hallmark feature of the disease is cerebral and cerebellar atrophy accompanied by enlargement of the lateral ventricles. All forms of NCL are currently fatal, with no curative therapies. Danon Disease is an X-linked dominant lysosomal storage disorder caused by pathogenic variants in the LAMP2 gene, which encodes the lysosome-associated membrane protein-2 (LAMP-2). Deficiency of the LAMP-2B isoform, which is highly expressed in the heart, skeletal muscle, and brain, is central to the disease mechanism. The classic phenotype is defined by a triad of cardiomyopathy, skeletal myopathy, and intellectual disability, most pronounced in affected men. Males typically present during adolescence, while females show later onset and a milder course, although cardiac involvement is common. The increasing availability of LAMP2 testing in cardiomyopathy gene panels has led to more frequent diagnoses. Genotype–phenotype correlations suggest that truncating mutations are associated with earlier and more severe disease, whereas certain missense variants restricted to LAMP-2B may result in a milder phenotype. Current management is symptomatic and transplant-oriented, but future therapies are directed toward gene-based correction and lysosomal pathway modulation [82].

Lastly, the spectrum of LSDs is further widened by conditions like RNASET2-Deficient Leukoencephalopathy. It is an autosomal recessive disorder caused by loss-of-function mutations in the RNASET2 gene, encoding a lysosomal T2 family acidic endoribonuclease responsible for degradation of rRNA within lysosomes. Pathophysiologically, defective RNASET2 results in accumulation of undegraded RNA in neurons, triggering innate immune activation (notably of interferon-stimulated genes), neuroinflammation, impaired microglial clearance of apoptotic debris, white matter lesions (including cystic/temporal lobe involvement), and progressive neurodegeneration. Clinically, infants present with severe psychomotor delay, seizures, hypotonia or spasticity, microcephaly, neurological regression, and white matter changes on MRI that can resemble congenital CMV infection or Aicardi–Goutières syndrome. Recent animal model studies suggest that microglia/macrophage-targeted therapies may offer a future treatment avenue beyond purely supportive care [83,84,85].

**Table 4:** Lysosomal disorders associated with cross-organelle interaction defects.

Cross-Organelle Interaction Defects	Gene(s)	Protein(s)	Clinical Features	Inheritance	Prevalence	References
Pompe Disease	GAA	Alpha glucosidase	Cardiomyopathy, hypotonia, respiratory distress, feeding intolerance, hepatomegaly, macroglossia, developmental delay, premature death.	Autosomal recessive	2.4: 100,000	[81]
Neuronal ceroid lipofuscinoses/ Batten disease	CLN 1, 2, 3, 4, 5, 6, 7, 8	Palmitoyl-protein thioesterase 1, DnaJ heat shock protein family (Hsp40) member C5, Tripeptidyl peptidase 1, CLN3 lysosomal/endosomal transmembrane protein, CLN5 intracellular trafficking protein, CLN6 transmembrane ER protein, major facilitator superfamily domain containing 8	Visual impairment, seizures, motor dysfunction, neuropsychiatric symptoms, dementia, dysphagia, motor regression, spasticity, myoclonus, epilepsy.	Autosomal dominant	No data available	[86]
Danon	LAMP2	Lysosomal-associated membrane protein 2	Cardiomyopathy, arrhythmias, myopathy, impaired motor skills, elevated CK, seizures, motor and coordination deficits, retinal and corneal pathology, hepatic and gastrointestinal involvement.	X-linked dominant	No data available	[82]
RNASET2-Deficient Leukoencephalopathy	RNASET2	Ribonuclease T2	Developmental delay, movement disorders, leukoencephalopathy, seizures, muscle rigidity.	Autosomal recessive	No data available	[83]

CK, Creatine Kinase. All protein names and gene symbols were curated and verified using the Human Protein Atlas database [64].

## 3. Genetic and Pathophysiological Complexity of Lysosomal Storage Disorders in the Era of Precision Medicine

The landscape of LSDs illustrates the intricate relationship between genetic mutations, disrupted lysosomal function, and the broad phenotypic spectrum observed in neurological diseases. LSDs, once classified as rare metabolic conditions, are increasingly recognized as central contributors to neurodegeneration, owing to their pivotal role in macromolecule degradation, autophagy, and cellular waste clearance. Similar to mitochondrial diseases, traditional diagnostic frameworks often fail to capture the underlying molecular heterogeneity of lysosomal disorders, limiting therapeutic precision [87]. In this setting, precision medicine does not simply provide a new label but functions as an essential tool for bridging molecular insights with clinical practice. By incorporating genomic sequencing, biochemical profiling, and phenotypic stratification, precision medicine enables earlier recognition of atypical presentations, identifies modifiers of disease progression, and informs treatment selection on an individual level [88]. Its value lies in translating genetic and mechanistic discoveries into practical interventions, ensuring that therapies such as enzyme replacement, substrate reduction, pharmacological chaperones, or gene therapy are matched to the patient most likely to benefit. For lysosomal disorders, this approach transforms a traditionally uniform outlook into a framework where prognosis and therapy can be tailored, ultimately improving clinical outcomes [89].

A complementary dimension to the evolving understanding of lysosomal biology is the adoption of functional classifications of LSDs, which move beyond substrate-based frameworks to organize disorders according to the lysosomal pathway that is impaired. This mapping distinguishes enzymatic hydrolytic defects, transporter-related disorders, biogenesis and signaling defects, and cross-organelle interaction abnormalities. By aligning disease categories with molecular mechanisms, this approach enhances the capacity of precision medicine to identify therapeutic entry points [90]. For instance, patients with enzymatic deficiencies may benefit from ERT or pharmacological chaperones, whereas transporter-related disorders such as cystinosis or NPC are better suited to SRT or transport-modulating strategies. Similarly, disorders of lysosomal biogenesis, exemplified by I-cell disease or Hermansky–Pudlak syndrome, highlight the need for interventions targeting enzyme trafficking and lysosomal assembly, while cross-organelle interaction defects emphasize the importance of therapies modulating autophagy and organelle crosstalk. Incorporating this functional perspective also provides a translational roadmap that connects genotype and pathophysiology to rational therapeutic design [91].

Advancements in genomic technologies, including whole-exome and whole-genome sequencing, have accelerated the identification of pathogenic variants in lysosomal genes such as GBA (associated with Parkinson’s disease), GALC (Krabbe disease), and GLA (Fabry disease) [92]. These tools facilitate earlier diagnosis, allow the detection of atypical phenotypes, and inform targeted interventions such as ERT, SRT, and chaperone-mediated therapies. For example, pharmacological chaperones like migalastat have demonstrated efficacy in stabilizing misfolded lysosomal enzymes in Fabry disease, illustrating how molecular characterization can guide therapeutic selection [93].

Therapeutic innovation in the lysosomal field is advancing rapidly, particularly in gene therapy and autophagy-modulating strategies. Recombinant AAV vectors are being deployed in clinical trials for several LSDs, including GM1 gangliosidosis and MLD, aiming to restore lysosomal function by delivering wild-type genes to affected tissues [94]. Moreover, genome-editing technologies, although still in early stages, show promise in addressing mutations in genes like NPC1, implicated in NPC. Lysosomal gene therapy, unlike mitochondrial approaches, benefits from well-characterized nuclear gene targets and more effective gene delivery to neural tissues. However, challenges such as immune responses, vector biodistribution, and the BBB persist.

Furthermore, lysosomal dysfunction has emerged as a key feature of complex neurodegenerative diseases such as Alzheimer’s, Parkinson’s, and frontotemporal dementia. In these disorders, impaired autophagy and endolysosomal trafficking contribute to protein aggregation, neuronal death, and synaptic dysfunction. The integration of multi-omics platforms—transcriptomics, proteomics, and lipidomics—with systems biology approaches can aid in identifying predictive biomarkers and constructing mechanistic disease models [95]. Future directions include the development of pharmacogenomic frameworks tailored to lysosomal transporters, enzyme variants, and drug uptake dynamics, alongside scalable gene-editing platforms and tissue-specific delivery vectors.

## 4. Conclusions and Prospects

In this review, we propose a cellular-pathway-based classification of lysosomal storage disorders, comprising four principal groups: enzymatic hydrolytic defects, transporter-related defects, biogenesis and signaling defects, and cross-organelle interaction abnormalities. This scheme was developed to accommodate the pronounced clinical and genetic heterogeneity of lysosomal disorders and to overcome the limitations of traditional substrate-based classification systems. By reframing these conditions according to the specific lysosomal pathways affected, our approach integrates genetic and mechanistic data into a unified conceptual model. The convergence of genetic evidence and pathway-based insights into lysosomal dysfunction offers a robust foundation for redefining how we understand and treat neurological disorders. Despite their clinical and genetic heterogeneity, lysosomal storage and related disorders share a core theme of disrupted degradation, recycling, and cellular clearance pathways—processes critical for neuronal survival and synaptic maintenance. Lysosomal diseases predominantly arise from nuclear gene mutations, simplifying gene therapy design but still presenting obstacles related to delivery and immune tolerance. Precision medicine strategies, grounded in genomic sequencing, proteomic profiling, and pathway-specific drug development, are increasingly being employed to tailor interventions in conditions like GD, Pompe disease, and NPC. Therapies such as enzyme replacement, pharmacological chaperones, and gene transfer techniques are progressing from preclinical models to human application, offering hope for disease modification rather than symptomatic relief.

Nevertheless, significant challenges remain, including the rarity of these diseases, limited access to the CNS, variable disease penetrance, the absence of solid biomarkers for treatment response, and the incomplete understanding of lysosome–organelle crosstalk within the CNS. Addressing these gaps will require investment in lysosome-targeted delivery systems, genome-wide functional screens, and collaborative clinical trials across genetically diverse populations. Bridging the gap between molecular pathogenesis and clinical intervention will enable the development of effective, personalized treatments that improve outcomes and quality of life for individuals affected by lysosomal impairment-related neurological disorders. Future research should integrate multi-omics profiling with functional lysosomal pathway analysis to enhance early diagnosis and guide precision therapeutic design. The translation of emerging gene- and nanomedicine-based strategies will depend on overcoming barriers to CNS delivery, ensuring long-term safety, and validating efficacy across genetically and phenotypically diverse patient populations.

## Data Availability

No new data were created or analyzed in this study. Data sharing is not applicable to this article.

## Abbreviations

AFD	Anderson–Fabry Disease
AGU	Aspartylglucosaminuria
AMRF	Action Myoclonus–Renal Failure
ARSA	Arylsulfatase A
ASM	Acid Sphingomyelinase
ASMD	Acid Sphingomyelinase Deficiency
ASOs	Antisense Oligonucleotides
BBB	Blood–Brain Barrier
BMT	Bone Marrow Transplantation
CHS CK	Chediak–Higashi Syndrome Creatine Kinase
CKD	Chronic Kidney Disease
CNS	Central Nervous System
ENT	Ear, Nose, and Throat
ERT	Enzyme Replacement Therapy
FGE	Formyglycine-generating Enzyme
GAGs	Glycosaminoglycans
Gb3	Globotriaosylceramide
GD	Gaucher Disease
GI	Gastrointestinal
GluCer	Glucosylceramide
GluSph	Glucosylsphingosine
GNS	N-acetylglucosamine-6-sulfatase
GSL	Glucosphingolipid
GST	Gene Silencing Therapy
HexA	β-Hexosaminidase A
HGSNAT	Acetyl-CoA:α-glucosaminide N-acetyltransferase
HLH	Hemophagocytic Lymphohistiocytosis
HSCT	Hematopoietic Stem Cell Transplantation
I2S	Iduronate-2-sulfatase
LIMP-2	Lysosomal Integral Membrane Protein-2
LNPs	Lipid Nanoparticles
LRO	Lysosome-Related Organelle
LSDs	Lysosomal Storage Disorders
Lyso-Gb3	Globotriaosylsphingosine
ML II	Mucolipidosis Type II
ML IV	Mucolipidosis Type IV
MLD	Metachromatic Leucodystrophy
MPS I	Mucopolysaccharidosis type I
MPS II	Mucopolysaccharidosis type II
MPS III	Mucopolysaccharidosis type III
MPS VI	Mucopolysaccharidosis type VI
MSD	Multiple Sulfatase Deficiency
NAGLU	α-N-acetylglucosaminidase
NPC	Niemann–Pick Disease Type C
NPD	Niemann–Pick Disease
PNS	Peripheral Nervous System
PPCA	Protective Protein/Cathepsin A
SAPs	Sphingolipid Activator Protein Deficiencies
SGSH	Heparan N-Sulfatase
SRT	Substrate Reduction Therapy
α-GalA	α-Galactosidase A
α-NAGA	α-N-acetylgalactosaminidase
β-GC	β-glucocerebrosidase
